# Vaginal microbiome dysbiosis and sexually transmitted infections correlate with concentrations of immunoglobulin isotypes in human cervicovaginal mucus: insights into HIV-1 transmission

**DOI:** 10.3389/fimmu.2025.1627807

**Published:** 2025-07-04

**Authors:** Matrona M. Akiso, Israel Abook, Marianne W. Mureithi, Janet Kombo, Print Koi, Joel Musando, Ruth J. Chirchir, Michael D. McRaven, Ann M. Carias, Sarah Joseph, Omu Anzala, Thomas J. Hope

**Affiliations:** ^1^ Department of Medical Microbiology and Immunology, Faculty of Health Sciences, University of Nairobi, Nairobi, Kenya; ^2^ KAVI – Institute of Clinical Research, Faculty of Health Sciences, University of Nairobi, Nairobi, Kenya; ^3^ Feinberg School of Medicine, Cell and Developmental Biology Department, Northwestern University, Chicago, IL, United States; ^4^ Department of Infectious Disease, Faculty of Medicine, Imperial College London, London, United Kingdom

**Keywords:** cervicovaginal mucus, immunoglobulins, HIV-1, bacterial vaginosis, STIs

## Abstract

**Introduction:**

Little is known about the relationship between antibody isotype in cervicovaginal mucus (CVM) and the local microenvironment and how this impacts HIV-1 transmission at the female genital mucosa.

**Methods:**

In a cohort of 139 adult women in Kenya, we measured antibody isotypes in CVM and describe their associations with local pH, serum concentrations of estrogen and progesterone, and sexually transmitted infections (STIs), including HIV-1.

**Results:**

We found that immunoglobulin G2 (IgG2) was the most abundant and IgG4 was the least abundant in the CVM. Overall, IgG1 concentrations were significantly lower in CVM samples from women with bacterial vaginosis (BV) compared to those without BV. Among women with BV, IgG1 concentrations declined further as vaginal pH increased, suggesting possible pH-mediated degradation. We also report negative associations of BV status with IgG3 and IgG4. In addition, infection with *Mycoplasma genitalium* and *Neisseria gonorrhoeae* was positively associated with concentrations of IgA and IgM, respectively. We also found the relationship between antibody isotype and subclasses with HIV-1 viral mobility *in vitro*. IgG3 concentrations negatively correlated with CAP045 HIV-1 mobility and IgG1 concentrations negatively correlated with the mobility of the 92TH023 recombinant HIV-1 strain upon VRC01 depletion. These observations point towards a potentially protective role for IgG1 and IgG3 in trapping certain HIV-1 strains in the CVM.

**Discussion:**

Importantly, our study builds on previous work, providing a potential mechanism by which BV and STIs may modulate immunoglobulin isotype and subclass content in the CVM. These results highlight the need for proper treatment of BV and other STIs, as this could impact the effectiveness of HIV-1 vaccines targeted at enhancing specific immunoglobulin responses in the cervicovaginal mucosa.

## 1 Introduction

Humoral immunity undoubtedly contributes to the barrier function of the female reproductive tract (FRT) mucosa and defense against sexually transmitted infections (STIs) including human immunodeficiency virus type 1 (HIV-1). For example, a previous study using undiluted cervicovaginal mucus (CVM) demonstrated consistent trapping of HIV in samples deliberately spiked with broadly neutralizing antibodies (VRC01, PGT121, and 2F5) (1). Another study showed that endogenous and exogenously added herpes simplex virus type 1 (HSV-1)-specific immunoglobulin G (IgG) was able to trap HSV-1 virions in fresh and pH neutralized CVM samples *in vitro* (2). In the same study, glycoprotein G (gG)-specific IgG1 significantly protected mice from HSV-2 infection and reduced the average viral load after vaginal challenge and infection with two doses of HSV-2 (2). Additionally, although not in the FRT, the importance of mucosal humoral immune responses has also been illustrated in the context of coronavirus. A study in severe acute respiratory syndrome coronavirus 2 (SARS-CoV-2) convalescent individuals demonstrated that induction of humoral immunity in the nasal mucosa was responsible for the reduction of viral shedding post-infection and may mediate protective immunity following re-exposure, thereby postulating that elevated nasal neutralization activity was associated with milder symptoms (3). Along similar lines, immunoglobulins have also been shown to directly immobilize bacteria in mucus, limiting their access to target cells and facilitating faster clearance from mucosal surfaces. Lipopolysaccharide (LPS)-binding IgG was able to arrest highly motile *Salmonella typhimurium* in mouse gastrointestinal mucus, markedly reducing *Salmonella* penetration (4). This ability of IgG to impact the mobility of not only viruses, but also a highly motile bacterium, further illustrates the effectiveness of humoral immunity (4–6). Vaccines targeting mucosal humoral immune responses could therefore be of great importance in controlling mucosal pathogen transmission and disease progression.

However, one understudied area concerning humoral immunity, especially at the FRT mucosa, is how tissue local immunoglobulin isotypes and subclass impact potential effectiveness. Variabilities in the content of the different immunoglobulin isotypes and subclasses may be influenced by both the local mucosal microenvironment and other physiological factors such as the menstrual cycle. A previous study utilizing cervicovaginal lavage (CVL) samples from the CAPRISA 004 and CAPRISA 008 clinical trial studies demonstrated positive correlations between concentrations of the different mucosal immunoglobulin isotypes and subclasses with the genital inflammation (7). Although this study analyzed CVL samples where dilution factors are variable and not well-defined during sample collection, the data demonstrate the impact of local mucosa microenvironment on the concentrations of the different immunoglobulin isotypes and subclasses. This could therefore elicit different effector mechanisms of the different immunoglobulin isotypes and subclasses against different pathogens. For instance, two independent *in vivo* studies utilizing rhesus monkeys demonstrated isotype-dependent variability in the effectiveness of different monoclonal neutralizing antibodies as protection against infection with intrarectal R5 SHIV challenge with dimeric IgA1 (dIgA1) offering the best protection compared to dIgA2 and IgG1, of which the latter two were comparable (8, 9). Nevertheless, there remain limited data on the relative concentrations of the different immunoglobulin isotypes and subclasses in human CVM and their associations with the cervicovaginal microenvironment, host physiologic factors, and infection with STIs, including HIV-1. A deeper understanding of the composition of immunoglobulins at genital mucosal surfaces and their regulation could aid the design of effective therapeutic and prevention strategies against HIV-1 transmission, where neutralizing antibodies are thought to play a crucial role. In this study, we characterized the immunoglobulin profile of specifically IgG1, IgG2, IgG3, IgG4, IgA, and IgM in CVM samples collected from adult Kenyan women. We examined associations with vaginal pH, serum hormonal concentrations, bacterial vaginosis (BV), STI status, and the mobility of transmitted/founder HIV-1 virions *in vitro*. These data aim to provide mechanistic insights into the mucosal factors that may modulate HIV-1 transmission risk in women.

## 2 Methodology

### 2.1 Participant recruitment

This study was approved by the Kenyatta National Hospital/University of Nairobi Ethics and Review Committee (protocol number P203/03/2022). All the study volunteers signed an informed consent form prior to their enrolment into the study. The study recruited 139 adult women (70 participants with HIV-1 and 69 participants without HIV-1) aged between 18 and 50 years within Nairobi County in Kenya. Of the women with HIV-1, 68 had been on antiretroviral therapy for an average of 89 months and 2 did not provide information on their treatment status. The study excluded pregnant women, women who were currently on or within 3 days of the end of their menstrual period, women who have had either protected or unprotected vaginal intercourse within the past 24 h, women on antibiotics within the past 3 days, or women with any condition that could have precluded provision of informed consent, compromised the volunteer’s health during the conduct of this study, or interfered with achieving the study objectives.

### 2.2 HIV-1 viral load analysis

HIV-1 viral load analysis was performed for the participants with HIV-1 using a GeneXpert machine (GeneXpert machine Dx system, Cepheid) according to the manufacturer’s instructions. Briefly, venous whole blood collected in a 4-mL K3 BD EDTA vacutainer tube (BD vacutainer^®^) was spun at 1,800 × *g* for 10 min at room temperature. One milliliter of plasma was then pipetted into a GeneXpert HIV-1 viral load cartridge (GeneXpert^®^ technology) and loaded into the GeneXpert machine for a run of approximately 2 h. The HIV-1 viral load was displayed in copies/mL of plasma with the limit of detection on the GeneXpert machine being 20 copies/mL and the limit of quantification being 40 copies/mL.

### 2.3 CD4 and CD8 count analysis

CD4 counts and CD8 counts were analyzed for the participants with HIV-1 using the BD FACS Count instrument according to the manufacturer’s instructions. Briefly, venous whole blood collected in a 4-mL K3 BD EDTA vacutainer tube (BD vacutainer^®^) was mixed well by inverting the tube repeatedly. The mixed blood (50 µL) was pipetted into each tube of the reagent tube pair tab (one tube for CD4 PE/CD3 PE-Cy™5 analysis and the second tube for CD8 PE/CD3 PE-Cy5 analysis) previously vortexed for approximately 5 s downwards and 5 s upwards. This was incubated for 1 h at room temperature and in the dark upon vortexing for 5 s. Fixative solution [50 µL; 5.0% formaldehyde and 1.76% methanol in 1× phosphate-buffered saline (PBS)] was then added into each of the tubes and the samples were vortexed for 5 s prior to running the tubes on the BD FACS Count instrument within 48 h of preparation.

### 2.4 CVM sample collection and process

CVM samples were collected by a trained study nurse/clinician using a softcup (Softcup^®^) following insertion into the vaginal vault for 1 h. The softcup was removed and put in a 50-mL falcon tube and placed on ice for transportation to the laboratory within 2 h. The CVM sample was then centrifuged at 1,160 × *g* for 10 min at 4°C immediately upon receipt in the laboratory. The supernatant CVM was transferred into a 1.5-mL microcentrifuge tube via a positive displacement pipetting system. Samples were then centrifuged again at 17,000 × *g* for 15 min at 4°C to separate the clear mucus from other cervicovaginal secretions. Approximately 90 µL of the supernatant (clear CVM) was then transferred into a clean 2-mL vial and stored at 4°C for later use in the viral mobility assay. The pH was measured using a 5-µL aliquot on the Baker-pHix Universal pH Indicator Stick (Avantor - J.T.Baker^®^, Germany). The remaining samples (both supernatant and pellet) were mixed with an equal volume of 1**×** protease inhibitor and mixed well by pipetting up and down several times. The samples were centrifuged again and 2 aliquots of the supernatant (50 µL each) were put in 2-mL vials for storage. Both the supernatant aliquots and the remaining pellet sample were frozen at −80°C.

### 2.5 Immunoglobulin isotyping assay

Concentrations of CVM IgA, IgG1, IgG2, IgG3, IgG4, and IgM were assessed using a Bio-Rad Bio-Plex™ Pro Human Isotyping Assays kit according to the manufacturer’s instructions. Data were acquired on a Luminex 200 reader with the immunoglobulin concentration reported in nanograms per milliliter upon interpolation from the standard curve concentrations (ng/mL) and the median fluorescence intensities. Intra-assay precision was calculated from the median fluorescence intensity of standard dilution points from duplicate wells with an acceptable coefficient of variation of less than 20%. Inter-assay precision was calculated from the median fluorescence intensity of standard dilution points from three different plates with an acceptable coefficient of variation of less than 20% (**Supplementary Figure 1**).

### 2.6 Bacterial vaginosis diagnosis

A cotton swab was used to swab the vaginal walls of a participant and a smear was made on a glass slide. Gram staining procedure was performed as per Smith and Hussey’s protocol (10). Air-dried, heat-fixed smear of cells on a glass slide was flooded for 1 min with crystal violet staining reagent before washing the slide in a gentle and indirect stream of tap water for 2 s. The smear was then flooded with a mordant, Gram’s iodine, for 1 min followed by washing in a gentle and indirect stream of tap water for 2 s. Next 95% (vol/vol) ethanol was added drop by drop to the slide to decolorize it before flooding the slide with a counterstain, safranin, for 30 s. The slide was then washed in a gentle and indirect stream of tap water until no color appeared in the effluent and then blot dried with an absorbent paper. The slide was examined under oil immersion using a brightfield microscope. BV diagnosis was done using the Nugent scoring system as proposed by Robert P. Nugent in 1991 where a score of 0 to 3 is considered BV negative, a score of 4 to 6 is considered intermediate, and a score of 7 or higher is considered BV positive (11).

### 2.7 Deoxyribonucleic acid extraction

Deoxyribonucleic acid (DNA) was extracted from the frozen CVM pellet samples using the DNeasy^®^ Blood & Tissue kit (Qiagen). The samples were first equilibrated to RT and then mixed well. Two hundred microliters of this suspension and 10 µL of an internal control (IC) DNA provided in the Sacace Multiplex Real Time PCR kit (Sacace technologies) were added into a 1.5-mL centrifuge tube containing 20 µL of QIAGEN proteinase. Buffer AL (200 µL) was then added. This mixture was then pulse-vortexed for 15 s and incubated at 56°C for 10 min. Two hundred microliters of 96% ethanol was added to the sample and mixed by pulse-vortexing for 15 s. The mixture was carefully applied to the kit-provided DNeasy Mini spin columns placed in a 2-mL collection tube and centrifuged at 8,000 rpm for 1 min. The DNA was washed twice with consecutive addition of 500 µL of buffer AW1 and AW2 as per the manufacturer’s instructions. The DNA was eluted with 200 µL of elution buffer AE into a clean 1.5-mL tube.

### 2.8 Real-time PCR for the detection of other STIs (*Neisseria gonorrhoeae, Chlamydia trachomatis, Mycoplasma genitalium*, and *Trichomonas vaginalis*)

To test for infection with *Neisseria gonorrhoeae*, *Chlamydia trachomatis*, *Mycoplasma genitalium*, and *Trichomonas vaginalis*, a real-time multiplex PCR reaction was done using a multiplex real-time PCR kit for the detection of *N. gonorrhoeae, C. trachomatis, M. genitalium*, and *T. vaginalis* (Sacace technologies) following the manufacturer’s instructions: A 25-µL PCR reaction mix for each sample was prepared by the addition of 10 µL of PCR-mix-1-FL *N. gonorrhoeae*/*C. trachomatis*/*M. genitalium*/*T. vaginalis*, 5 µL of PCR-mix-2-FRT, 0.5 µL of polymerase (TaqF), and 10 µL of the DNA sample/positive control/negative controls. The data were acquired on a 6-plex rotor gene real-time PCR thermocycler. The amplification protocol had a 15-min hold at 95°C and two cycling sets. The first cycling set had five cycles of 95°C for 5 s, 60°C for 20 s, and 72°C for15 s. Fluorescence was recorded at the second step of cycle 2 having 40 cycles with the same temperature conditions as the first cycling set.

### 2.9 Progesterone and estrogen profiling in human serum

The hormones were measured using competitive chemiluminescent emission assay on an automated Cobas e 411^®^, a Roche 411 series as per the manufacturer’s instructions. Briefly, whole blood was collected in 5-mL BD serum separation tubes and approximately 1 mL of serum was obtained upon centrifugation at 1,800 × *g* for 10 min. Serum (500 µL) was pipetted into a sample cup and loaded into the Cobas analyzer, after which the concentrations of the hormones in the sample were obtained in picograms per milliliter.

### 2.10 HIV-1 viral diffusion in the human CVM

#### 2.10.1 Preparation and characterization of fluorescent HIV-1 particles

Fluorescently labeled HIV-1 virion particles were generated at the Hope laboratory, Northwestern University in Chicago, USA. Viruses were produced by polyethylenimine (Polysciences, Warrington, PA) co-transfection of a human embryonic kidney cell line (293T) with various proviral and fluorescent protein constructs. Briefly, 1 mL of 293T cell suspension from a confluent or nearly confluent 15-cm plate was seeded into a 10-cm plate for approximately 24 h to achieve 70% confluence. Proviral plasmids, based on availability, encoding either the clade B CH040 or R9 strain of Bal, clade C CAP045, or the circulating recombinant circulating form clade CRF01_AE 92TH023 viral envelopes, were then transfected into the 293T cells, along with a plasmid encoding the HIV-1 viral protein R (VPR) with a C-terminal mCherry fusion. Cells were washed with 1× phosphate-buffered saline approximately 24 h following transfection, fresh Dulbecco’s modified Eagle medium was added, and the culture was incubated for a 16-h period. The supernatant containing labeled viral particles was collected and concentrated 10-fold through a 20% sucrose cushion by ultracentrifugation at 28,000 rpm (~125,000 × *g*) and at 4°C in an SW 32 Ti rotor using a Beckman Optima X Series ultracentrifuge for 90 min. The p24 content of the virus preparations before and after ultracentrifugation was determined using a commercially available p24 enzyme-linked immunosorbent assay kit (PerkinElmer, Waltham, MA). Only viral preparations with a p24 concentration between 800 and 2,000 ng/mL based on this enzyme-linked immunosorbent assay were used. In addition, all virus preparations were assayed for infectivity using TZM-βl indicator cells. Finally, fluorescent protein incorporation was assessed by measuring co-localization of HIV-1 capsid protein and the given VPR fluorescence by staining of virions bound to coverslips with a monoclonal antibody against viral capsid protein AG3.0. Only viral preparations that contained at least 85% incorporation of the fluorescent VPR were used in experiments.

#### 2.10.2 Synthesis and characterization of PEGylated beads

MES sodium salt, *N*-(3-dimethylaminopropyl)-*N´*-ethylcarbodiimide hydrochloride (EDAC), and 2 -kDa α,ω-diamino polyethylene glycol (PEG) were purchased from Sigma-Aldrich (St. Louis, MO). Carboxylated nanoparticles with a diameter of 200 nm with excitation at 660 nm and emission at 690 nm (81 µmol CO**_2_** H/g) were purchased from Invitrogen. Pluronic F-127 was purchased from Spectrum Chemicals (Gardena, CA). To conjugate the α,ω-diamino PEG, 120 µL of MES buffer (pH 6.5) was added to 4 mL of an aqueous suspension of 5 mg/mL carboxylated nanoparticles (1.6 µmol of RCO2H = 1 eq). Next, 24 mg of α,ω-diamino PEG (2,000 g/mol, 12 µmol, 7.5 eq) was added as a solid and dissolved. Pluronic F-127 (20 mg/mL) was then added to a concentration of 240 μg/mL. The pH was adjusted between 6.5 and 7 with 1 M hydrochloric acid. Next, a 100-mg/mL solution of EDAC was freshly prepared in deionized water and used immediately. To the reaction, 46 µL of the EDAC solution (24 μmol, 15 eq) was added, and the reaction was incubated in a bath sonicator for 2 h at 5°C in the dark. A second coupling was performed with a second aliquot of 24 mg of α,ω-diamino PEG, pH adjustment between 6.5 and 7, and another 46 µL of a second freshly prepared 100 mg/mL EDAC solution. The nanoparticles were purified by ultracentrifugation using 10-kDa cutoff centrifugal filters (Millipore Amicon, Billerica, MA) by washing five times with deionized water until the filtrate was absent of soluble amine by the ninhydrin test. The particles were characterized by particle size analysis and zeta potential using a Zetasizer Nano S (Malvern Instruments, Westborough, MA). Zeta potential was measured at various pH values and calculated using the Hückel approximation.

#### 2.10.3 Mobility assays

We assessed virions or bead mobility in CVM using an established protocol at the KAVI Institute of Clinical Research laboratory (adapted from Prof. Thomas J. Hope laboratory, Feinberg School of Medicine, Cell and Developmental Biology Department, Northwestern University, Chicago, IL, USA). Briefly, a mixture of concentrated fluorescent virus, 200 nm of fluorochrome conjugated PEGylated beads, and the human CVM was made in a ratio of 1:1:7. The 200-nm PEGylated beads were used to control for the CVM viscosity. PEGylation reduces the adhesion of the beads to the mucin fibers in the CVM samples and, therefore, their mobility speed depending only on the mucus viscosity. An adhesive spacer was applied on a glass slide trimming any overhang. The virus/bead/mucus mixture (5 µL) was then put on the spacer and covered with a coverslip upon removal of the adhesive backing from the spacer. The edges of the covered glass slide were then sealed using a clear nail polish and allowed to dry for 30 min on ice. The sample was then imaged for particle movement using a deconvoluted fluorescent microscope available at KAVI-ICR. Each image was set at an exposure time of 150 ms and 60-s videos were recorded. Each sample had five videos recorded at different points. The particle motion in the CVM was calculated in terms of mean squared displacement (MSD) in µm/s using an IDL software using the Skynet program.

### 2.11 Statistical analysis

Comparisons among sample groups were made using the non-parametric Kruskal–Wallis test with Dunn’s multiple comparisons to compare the mean rank of each group with the mean rank of every other group. Non-parametric Mann–Whitney *U* test was used for comparison between sample groups while a multiple linear regression analysis was used to describe multiple associations. The multiple associations were adjusted for the participant’s age, serum concentrations of estrogen and progesterone, the CVM pH, BV, and STIs including HIV-1. Normality distribution was tested using the Shapiro–Wilk test while the level of significance was tested at 95% confidence interval (*p* < 0.05). All statistical analyses were performed on GraphPad Prism software version 10.4.2.

## 3 Results

### 3.1 Participant demographics

A total of 139 adult women were enrolled in the study, of whom 70 participants had HIV-1 and 69 participants had no HIV-1. Of the participants with HIV-1, 42 were negative for BV, 6 were intermediate, and 22 were positive for BV. In the group of participants who were negative for HIV-1, 41 were negative for BV, 6 were intermediate, 20 were positive, and 2 samples were not of sufficient quality to obtain a valid result. A total of 37 participants with HIV-1 and 34 participants without HIV-1 reported that they were using hormonal contraception while 33 participants with HIV-1 and 32 participants without HIV-1 were not on any contraceptive. One of the participants with HIV-1 and two of the participants without HIV-1 reported to be using condoms as a way of contraception. Four of the participants with HIV-1 and five of the participants without HIV-1 were positive for *N. gonorrhoeae*. A total of 11 participants with HIV-1 and 18 participants without HIV-1 were positive for *C. trachomatis* and 5 participants with HIV-1 and 4 participants without HIV-1 were positive for *M. genitalium*. A total of 20 participants with HIV-1 and 17 participants without HIV-1 tested positive for *T. vaginalis* (**Table 1**). Of the participants with HIV-1, 43 had undetectable viral load (<20 copies per mL), 19 had detectable but unquantifiable viral load (between 20 and 40 copies per mL), while 8 had detectable and quantifiable viral load with a median of 236 viral copies per mL of plasma. Of the participants with HIV-1, 68 were on antiretroviral treatment for a median time of 87.5 months. One participant with HIV-1 reported to have defaulted from treatment while one did not give any information on her treatment status. The median CD4 and CD8 counts for the participants with HIV-1 were 709 and 727, respectively (**Table 1**).

**Table 1:** Participant demographic data.

N	HIV positive	HIV negative
	70	69
Mean age in years (minimum–maximum)	35 (20–45)	32 (18–49)
Bacterial vaginosis (BV) status			
BV negative	42	41
BV intermediate	6	6
BV positive	22	20
Contraceptive use			
On hormonal contraceptives	37	34
On non-hormonal contraceptive (condom use)	1	2
Not on any contraceptives	33	32
Positive for sexually transmitted infections			
*Neisseria gonorrhoeae*	4	5
*Chlamydia trachomatis*	11	18
*Mycoplasma genitalium*	5	4
*Trichomonas vaginalis*	20	17
Co-infections (at least two of the tested STIs)	9	9
Negative for the tested STIs	41	36
Viral load (copies/mL)		
Undetected	43	N/A
20–40 (detected but unquantifiable)	19	N/A
>40 (Detected and quantifiable) median (25th percentile–75th percentile)	8 236.5 (126.5–3,485)	N/A
On ART treatment	68	N/A
Time on ART in months median (25th percentile–75th percentile)	87.5 (44.25–127.8)	N/A
Treatment defaulter	1	N/A
Data on ART treatment not available	1	N/A
CD4 counts median (25th percentile–75th percentile)	709 (560–1,014)	N/A
CD8 counts median (25th percentile–75th percentile)	727 (569.8–959.8)	N/A

N/A, not applicable.

### 3.2 IgG2 is the most abundant immunoglobulin subclass in CVM

**Figure 1:**
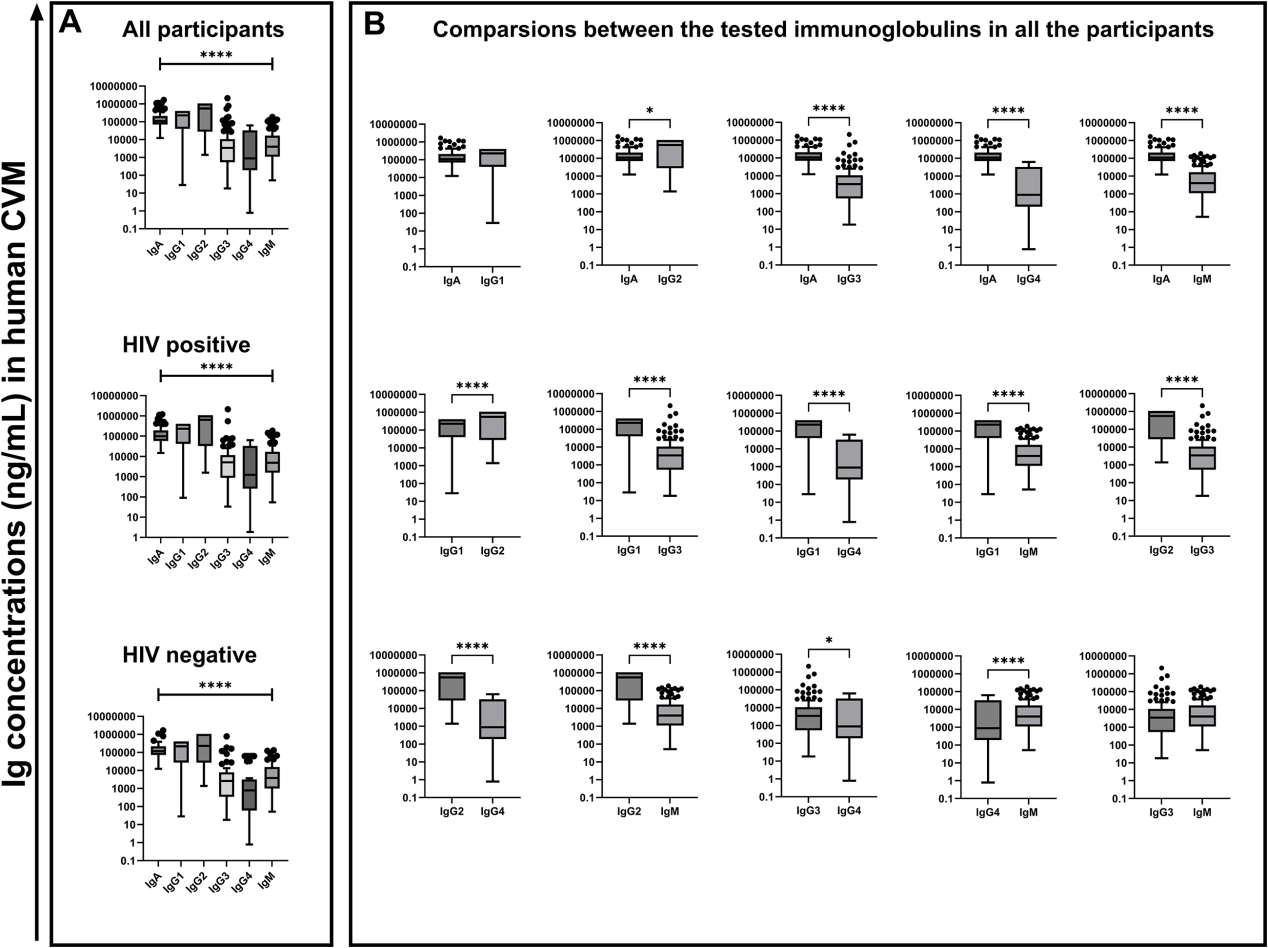
Concentrations of the different measured immunoglobulin subtypes (in ng/mL) in the human CVM of 139 participants. Statistical test used was the Kruskal-Wallis test **(A)** and Mann-Whitney *U* t test **(B)** at 95% confidence interval (CI) *p < 0.05, ****p < 0.0001, ns, not significant.

Immunoglobulin subtypes and subclasses have distinct biological effector functions; therefore, variations in the antibody composition in CVM may have an impact on the CVM barrier function. We therefore sought to assess immunoglobulin composition and concentration in the CVM using a Luminex assay utilizing a 6-plex kit for detection of six immunoglobulin isotypes/subclasses (IgA, IgG1, IgG2, IgG3, IgG4, and IgM). In general, there were significant differences (*p* < 0.0001) in the concentrations of the different immunoglobulin isotypes and subclasses in the CVM samples by Kruskal–Wallis test (**Figure 1A**). Upon comparison between each immunoglobulin concentration using a two-tailed Mann–Whitney *U* test, we observed that IgG2 had the highest concentrations in comparison to IgA (*p* = 0.0373) and in comparison to all the other immunoglobulins (*p* < 0.0001). The concentrations of IgA and IgG1 were comparable. However, both IgA and IgG1 concentrations were higher in comparison to the concentrations of IgG3, IgG4, and IgM (*p* < 0.0001). IgG3 concentrations were significantly higher compared to those of IgG4 (*p* = 0.0155). There was no difference between the concentrations of IgG3 and those of IgM. However, the concentrations of IgM were significantly higher relative to those of IgG4 (*p* < 0.0001) (**Figure 1B**). Differential CVM immunoglobulin isotype and subclass concentrations were subsequently shown to associate with clinically relevant variations in the local CVM microenvironment.

### 3.3 Bacterial vaginosis is negatively associated with IgG1 concentrations in human CVM

**Figure 2:**
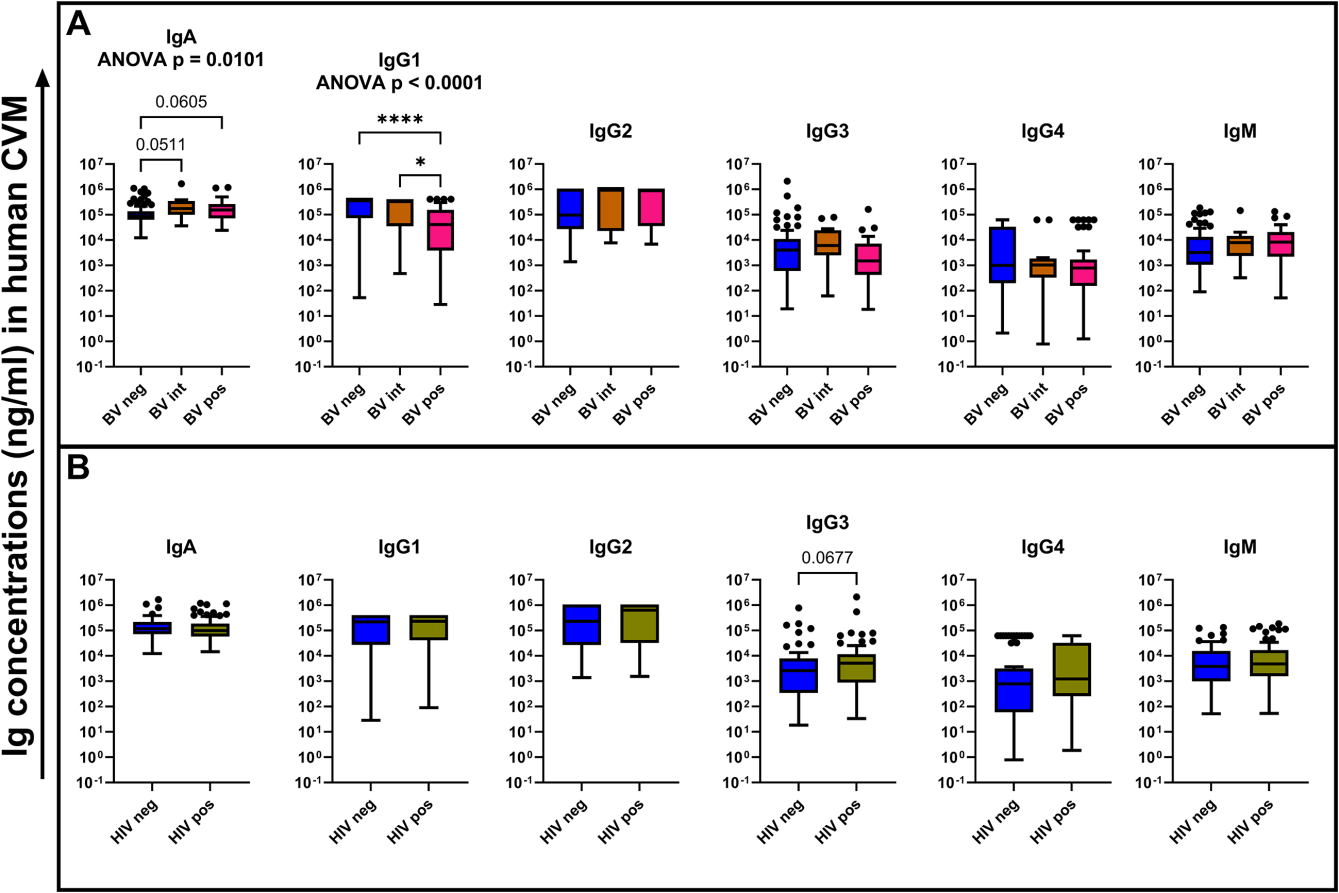
Comparison of the tested immunoglobulin concentrations (ng/mL) among the different BV status **(A)** and HIV-1 infection status **(B)**. Statistical tests used for comparison among the BV status was Kruskal–Wallis test with Dunn’s multiple comparison. For comparison between the HIV-1 infection status, a Mann–Whitney *U* test was used. The bars show the mean concentrations of the immunoglobulins while the error bars indicate the standard error of mean. *p < 0.05, ****p < 0.0001.

**Figure 3:**
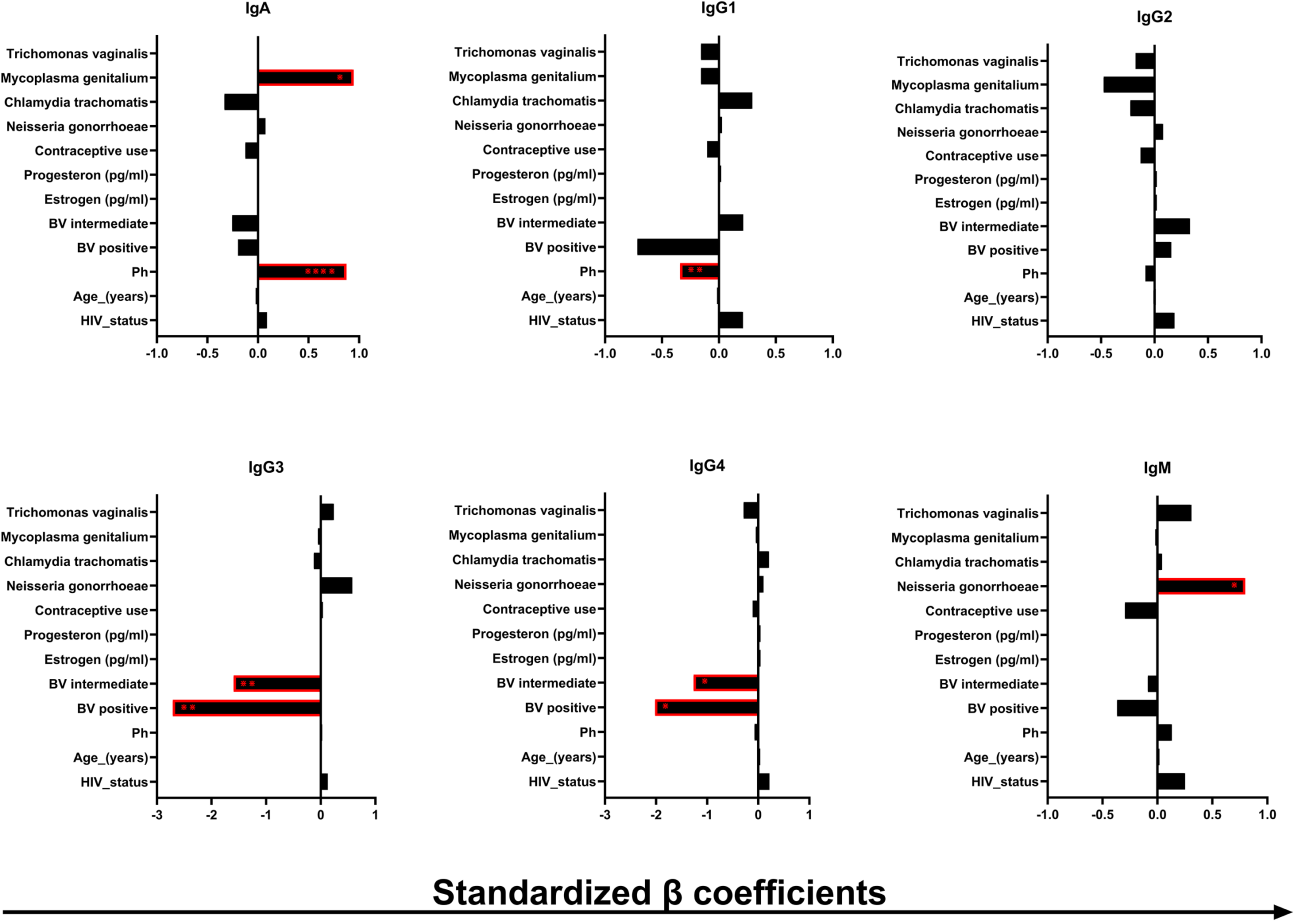
Association of the immunoglobulin concentrations (in ng/mL) in the human CVM with some of the microenvironmental factors in the human CVM. β coefficient and the *p* values were obtained from multiple linear regression analysis. The bars indicate the negative (left) and the positive (right) association β coefficient values. *p < 0.05, **p < 0.01, ****p < 0.0001.

The FRT mucosa is the primary portal of entry for STIs including HIV-1. The microbiome at this pivotal site plays an important role in shaping protective barrier properties as microbial dysbiosis has been associated with increased risks of HIV-1 acquisition (12). As immunoglobulins form part of the CVM protective component, we sought to assess the effect of microbial dysbiosis on the abundance of the measured immunoglobulin isotypes and subclasses among the BV status across the whole cohort using the Kruskal–Wallis test with Dunn’s multiple comparisons test. We found that CVM IgG1 concentrations were significantly lower in BV-positive samples relative to BV-negative (*p* < 0.0001) and BV-intermediate (*p* = 0.0291) samples (**Figure 2A**). This was independent of HIV status but negatively associated to the CVM sample pH (**Figure 3**). This finding suggests an increased susceptibility of IgG1 degradation under BV conditions, which may be associated with increased concentrations of degradative enzymes or from elevated pH environments associated with BV. Additionally, there were trends towards increased IgA concentrations in BV-intermediate (*p* = 0.0511) and BV-positive (*p* = 0.0605) CVM samples compared to BV-negative samples, suggesting a protective role in response to the microbial dysbiosis (**Figure 2A**). We also noted a trend towards higher concentrations of IgG3 in CVM samples from participants with HIV-1 (*p* = 0.0677) relative to the samples from participants without HIV-1. Although this is not statistically significant in our results, it may suggest a possible elevation of IgG3 expression in HIV-1 infection, which may be due to HIV-1-associated hypergammaglobulinemia (13) (**Figure 2B**). We did not see any difference in the concentrations of the tested immunoglobulins across the CVM samples from women with any of the tested STIs relative to those without any of the tested STIs (**Supplementary Figure 2**).

### 3.4 Immunoglobulin concentrations in human CVM may be independently modulated by some STIs, microbial dysbiosis, and the CVM pH

The cervicovaginal local microenvironment and some host factors could potentially impact the presence and abundance of the different immunoglobulin isotypes and subclasses in the CVM. To understand these nuances better, we performed a multiple linear regression analysis to assess the potential impact of factors that could affect immunoglobulin concentrations in CVM samples, including participant’s age, use of contraceptives, HIV-1, BV and other STI status, pH, and concentrations of serum estrogen and progesterone (**Figure 3**). The β coefficient values were standardized by dividing the unstandardized values with the standard deviation of the data points from the predicted values (denoted as Sy.x in the GraphPad Prism regression model). The unstandardized and standardized β coefficient values are shown in **Supplementary Tables 1, 2**. Of the data combined, we found significant, independent positive associations between IgA and *M. genitalium* infection (*p* = 0.0197, β = 0.946) and pH (*p* ≤ 0.0001, β = 0.875). pH was also negatively associated with the concentrations of IgG1 (*p* = 0.0104, β = −0.341). In section 3.3, we showed decreased concentration of IgG1 in BV-positive CVM samples relative to the BV-intermediate and -positive samples. However, no associations between the IgG1 concentrations with BV were detected, but rather only negative associations with CVM pH. This observation suggests that CVM pH may have more impact in the modulation of IgG1 concentrations in CVM as opposed to the increased concentrations of degradative enzymes associated with BV. We also found that being BV intermediate or positive was independently negatively associated with IgG3 (*p* = 0.0066, β = −1.595 and *p* = 0.0013, β = −2.710, respectively) and IgG4 (*p* = 0.0302, β = −1.266 and *p* = 0.0156, β = −2.0200, respectively). The negative associations of IgG3 and IgG4 with BV suggest possible degradation of these immunoglobulins with BV-associated degradative enzymes. IgM was positively associated with *N. gonorrhoeae* infection (*p* = 0.0408, β = 0.7992), suggesting possible induction of IgM expression by *N. gonorrhoeae* infection. Our results suggest modulations of the humoral immunity in the CVM by the local microbial dysbiosis and STIs.

### 3.5 Hindrance to HIV-1 viral mobility in human CVM varies across different strains

**Figure 4:**
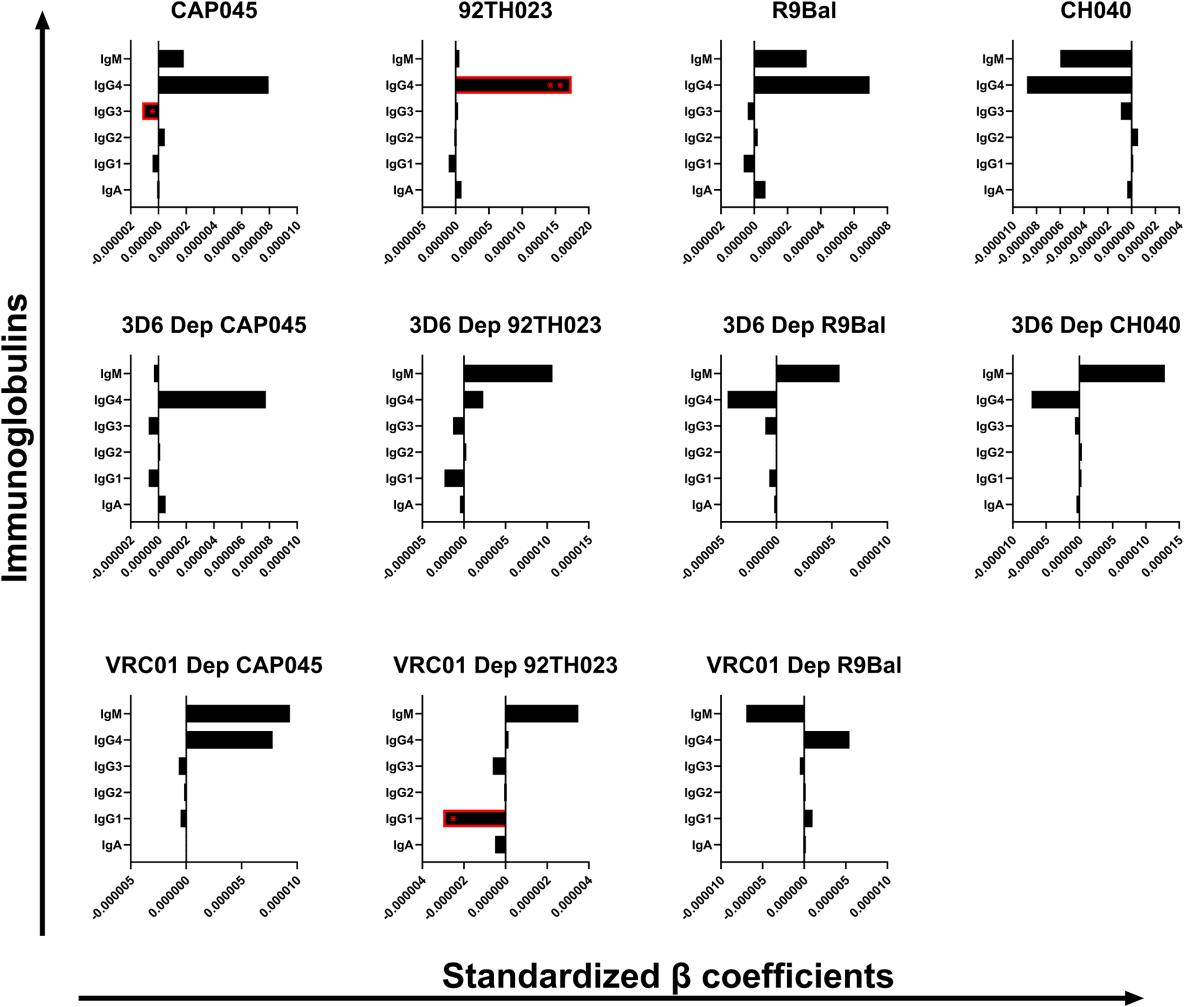
Association of the immunoglobulin concentrations (ng/mL) in the human CVM with the mobility of the tested T/F HIV-1 clades in the human CVM. β coefficients and *p*-values were obtained from multiple linear regression analysis. The error bars indicate the lower (left) and the upper (right) 95% confidence intervals of the β coefficient value. *p < 0.05.

Immunoglobulins in CVM work by binding pathogens and potentially limiting their motion and accessibility to their target cells (14). In addition, a previous study demonstrated that immunoglobulins may neutralize pathogens by binding to their epitopes and disabling the infection of their target cells (2). To better understand this phenomenon, we performed multiple linear regression analysis adjusted for HIV infection status, age, pH, BV status, concentrations of sex hormones, contraceptive use, and STIs to evaluate the relationships between the concentrations of the immunoglobulin isotypes and subclasses and the mobility of several T/F HIV-1 strains in CVM samples. The β coefficient values were standardized as explained in section 3.4. The unstandardized and standardized β coefficient values are shown in **Supplementary Tables 3, 4**. Although we did not notice associations between any of the tested immunoglobulins with the clade B T/F HIV-1 strain CH040 and the clade B laboratory adapted R9Bal, we did observe a significant negative association between concentrations of IgG3 and the mobility of the non-depleted clade C T/F HIV-1 CAP045 (*p* = 0.0419, β = −0.000001177). Additionally, we found that IgG1 concentrations had a negative correlation with the VRC01 depleted set of the circulating recombinant clade 92TH023 (*p* = 0.0113, β = −0.000002997) (**Figure 4**). On the other hand, the mobility of the non-depleted set of the recombinant clade A/E 92TH023 virions had a positive association (*p* = 0.0086, β = 0.00001745) with the concentrations of IgG4 in the CVM samples (**Figure 4**). Our results indicate viral mobility hindrance in the CVM, possibly by immunoglobulin binding to different regions of the envelop glycoprotein, which may be strain dependent.

## 4 Discussion

To our knowledge, our study is the first to quantify IgG subclasses in human CVM samples. Some previous studies have measured immunoglobulin concentrations in vaginal secretions (7, 15). However, these studies either measured total IgG concentrations and/or utilized CVL samples where dilution factors are variable and not well defined in the sample collection process. Therefore, the composition of IgG subclasses in the CVM has not been characterized to date. We tried to address this gap by quantifying the different IgG subclasses in human CVM samples from adult women in Nairobi, Kenya. We report higher concentrations of IgG2 relative to the other IgG subtypes. Although some previous studies reported higher levels on IgG1 in cervicovaginal secretions relative to the other IgG subclasses (7, 15), the difference in the sampling methods and study population could have influenced our results. Additionally, IgG2 has previously been shown to play a role in protection against bacterial infections in the periphery (16, 17), which might account for its relative abundance in the CVM given the continuous exposure to bacteria at this site. From five women who were either BV intermediate/positive and/or HIV positive, we also noted higher concentrations of IgA in their CVM samples. Previous studies have demonstrated the protective and homeostatic role of IgA in the gut mucosa with IgA shown to coat a diverse range of commensal gut microbiota, promoting a host–commensal symbiotic relationship and reducing/preventing pathogenic bacterial virulence (18–20). The antibacterial role of IgA has also been suggested in a study by Hein et al. Here, they reported increased concentrations of IgA and IgG in cervical mucus plugs of pregnant women relative to cervical mucus plugs from non-pregnant women. In addition, they reported higher bacterial loads in the vaginal side of the cervical mucus plugs relative to the uterine side, suggesting a possible gatekeeping role for IgA and IgG (21). It is possible that the increased concentrations of IgA in the BV intermediate/positive samples we report in our study are a response to the microbial dysbiosis and the increased concentrations of pathogenic bacteria.

In this study, we also describe a positive correlation between concentrations of IgA and *M. genitalium* infection, suggesting a possible role for this isotype in the response to this STI. Our results may therefore explain previous findings that demonstrated significantly higher concentrations of *M. genitalium-*specific IgA in cervical and vaginal samples of women with *M. genitalium* relative to women without *M. genitalium* (22). We also observed elevated concentrations of IgG3 in three CVM samples, of which two out of three were STI positive: one positive for HIV-1, *N. gonorrhoeae*, and *C. trachomatis* and another positive for HIV-1 and *T. vaginalis*. This is important as IgG3 has been previously associated with improved control of HIV-I infection, demonstrating more neutralization potency and improved Fc effector function of IgG3 compared to IgG1 (23). This could explain the elevated concentrations of IgG3 we observe in CVM samples from participants with HIV-1. We also found that infection with *N. gonorrhoeae* was positively associated with IgM concentrations. Although we have not identified previous studies directly evaluating the role of IgM in *N. gonorrhoeae* infection, it has previously been shown that a chimeric protein (C4BP-IgM), consisting of the two N-terminal gonococcal binding domains of complement inhibitor C4B protein with the Fc domain of IgM, restored susceptibility of *N. gonorrhoeae* to antibiotics through complement activation (24). This highlights an Fc-mediated protective role of IgM in some STIs, which could explain our observed positive correlation of this immunoglobulin subtype with *N. gonorrhoeae* infection. Although most HIV-1 vaccines aim at triggering production of neutralizing antibodies, observations from IgG3 and IgM Fc-mediated effector functions in some STIs provide additional avenues that, when harnessed, may aid in curbing HIV-1 transmissions and disease progression.

In contrast to the STIs that had positive associations with the concentrations of some immunoglobulins, dysbiosis in the vaginal microbiome had negative associations with some of the tested immunoglobulins. For instance, our results show significant reduction in IgG1 concentrations in BV-intermediate and BV-positive CVM samples when compared to the BV-negative samples, despite the HIV infection status. We also observe significant negative associations of IgG3 and IgG4 concentrations with BV-intermediate and BV-positive conditions. Similar to our results, a study by Liu et al. observed lower levels of total IgG and IgA in cervicovaginal secretions of women with BV in comparison to secretions from women who were intermediate or negative for BV (27). BV has been associated with increased activities of the degradative enzymes produced by the BV-associated bacteria such as the proteinases and glycosidases (28, 29). A previous study also showed that IgG heavy chain was more susceptible to proteolysis compared to secretory IgA and the proteolysis is even greater under BV conditions (30). Unlike in these two previous studies that analyzed total IgG, our study analyzed the different IgG subclasses, which hints more on which IgG subclass is possibly the most susceptible to BV-associated degradation. This is important as the presence and persistence of the different IgG subclasses may be differentially influenced by the vaginal microbiome potentially affecting the effectiveness of vaccines targeting the mucosal humoral immunity.

The menstrual cycle has been associated with variation in the CVM physical properties due to fluctuations in the concentrations of the reproductive hormones, estrogen and progesterone (31, 32). The use of hormonal contraceptives has also been associated with alterations in the physical properties of the CVM possibly by interfering with endogenous production of progesterone upon inhibition of ovulation (33). In addition, the use of progestin or estrogen containing oral contraceptives was significantly associated with increases in the cervical mucus mucins and a highly viscous mucus pattern in a study of women of reproductive age on oral contraceptives (34). All these alterations in the CVM physical properties could be associated with deterioration in the protective role of the CVM. As immunoglobulins form part of the CVM protective role, our study evaluated the correlations of the immunoglobulin concentrations to serum concentrations of estrogen and progesterone. Previous studies demonstrated that elevated concentrations of reproductive hormones were associated with increased concentrations of immunoglobulin-producing cells in the cervical mucus (35). Exogenous estrogen was associated with increased transportation of secretory immunoglobulins into the lumen upon culturing human endometrial cells with interferon gamma and interleukin 4 due to its influence on expression of the secretory component (36). We, however, did not observe any association between the concentrations of any of the tested immunoglobulins and the concentrations of reproductive hormones (**Figure 3**). Similarly, there was no association in the concentrations of any of the tested immunoglobulins with the use of contraceptives (**Figure 3**). We did not also observe any difference in the concentrations of the tested immunoglobulins between CVM samples from participants who used progestin-only contraceptives and those who used combined (estrogen and progestin containing) contraceptives (**Supplementary Figure 3**). This observation could have been influenced by our cross-sectional study design where samples were collected only at one time point across the menstrual cycle from each participant.

Ideally, an HIV-1 vaccine would induce binding and/or neutralizing antibodies at mucosal portals of entry such as the cervix. Work by Schaefer et al. demonstrated that several broadly neutralizing antibodies (VRC01, PGT121, and 2F5) are able to reduce the mobility of HIV pseudo-viruses in CVM *in vitro* and that the effect was modulated by the local microbiome (1). We assessed the correlation between the concentrations of the immunoglobulins in the CVM samples to the mobility of selected clades of T/F HIV-1 variants in the CVM. We observed significant negative correlations between the concentrations of IgG3 with CAP045 mobility and IgG1 with recombinant 92TH023 mobility. As previously mentioned, IgG3 has been associated with neutralization potency and improved Fc effector function (23). This improved neutralization potency has been associated with the longer hinge length of IgG3 (37). Exploring this property to genetically engineer the other IgG subclasses could enhance their neutralization potencies and increase the breadth of HIV-1 neutralization. Our results further suggest hindrance to the mobility of the recombinant HIV-1 strain 92TH023 possibly by binding of IgG1 to the gp41 stump as demonstrated by the negative association of IgG1 to the mobility of the VRC01-depleted 92TH023. Recombination is one of the immune escape mechanisms by HIV-1, resulting in more resistant strains (38). Our findings, therefore, suggest the possible exposure of epitopes that are only recognized by binding antibodies through the 92TH023 recombinant HIV-1 strains, and this could be a way to evade neutralization.

This study had some limitations. The cross-sectional study design meant that we were unable to associate temporal variations in the menstrual cycle and associated hormonal changes with the variations in immunoglobulin concentrations within the same individual, potentially resulting in inter-personal differences. While we have described significant associations between BV and immunoglobulin isotype, more detailed characterization of the microbiome was beyond the scope of this study, and we were unable to further resolve any relationships between specific microbial communities such as *Gardnerella* and *Prevotella* and variations in immunoglobulin isotypes. Limitations in the sample size precluded statistically meaningful analysis when numbers were small, which was true for certain STIs (such as *M. genitalium* and *C. trachomatis*). This could have influenced our results when comparing immunoglobulin concentrations between women who were positive for any of the tested STIs and those who were negative for the tested STIs. We were unable to test for human papillomavirus (HPV) and HSV in this study and cannot rule out the role of these pathogens in variations in immunoglobulin isotypes. For example, a study in vaginal washes of women who were positive for HPV DNA observed the presence of secretory IgA specific to the HPV16 capsid antigens (25). In addition the presence of IgA and IgG specific to HSV-1 and HSV-2 have been shown in cervicovaginal secretions of women with evidence of HSV-2 infection (26). The two studies demonstrate the possible influence of other STIs on the concentrations of the different immunoglobulins in the CVM. Lastly, observations from the single population in Kenya may not be generalized globally. We therefore recommend future longitudinal studies across different populations and looking at different time points across the menstrual cycle. We also recommend the evaluation of other STIs and the microbiome composition and variations across the menstrual cycle and the possible associations with CVM immunoglobulin concentrations.

In conclusion, this study highlights the impact of microbial dysbiosis and elevated pH on mucosal immunoglobulin composition, which may influence susceptibility to HIV-1 transmission. These findings underscore the need for prompt BV and STI treatment and consideration of mucosal immunity in HIV vaccine strategies.

## Data Availability

The raw data supporting the conclusions of this article will be made available by the authors, without undue reservation.
