# Supplementary material for: Vaginal microbiome dysbiosis and sexually transmitted infections correlate with concentrations of immunoglobulin isotypes in human cervicovaginal mucus: insights into HIV-1 transmission
Source: Front Immunol. 2025 Jul 4;16:1627807. doi: 10.3389/fimmu.2025.1627807 (PMC12271226; doi:10.3389/fimmu.2025.1627807)
Supplement: Supplementary file 1 [file DataSheet1.docx]

Supplementary Material

# Supplementary Figures and Tables

## Supplementary Figures


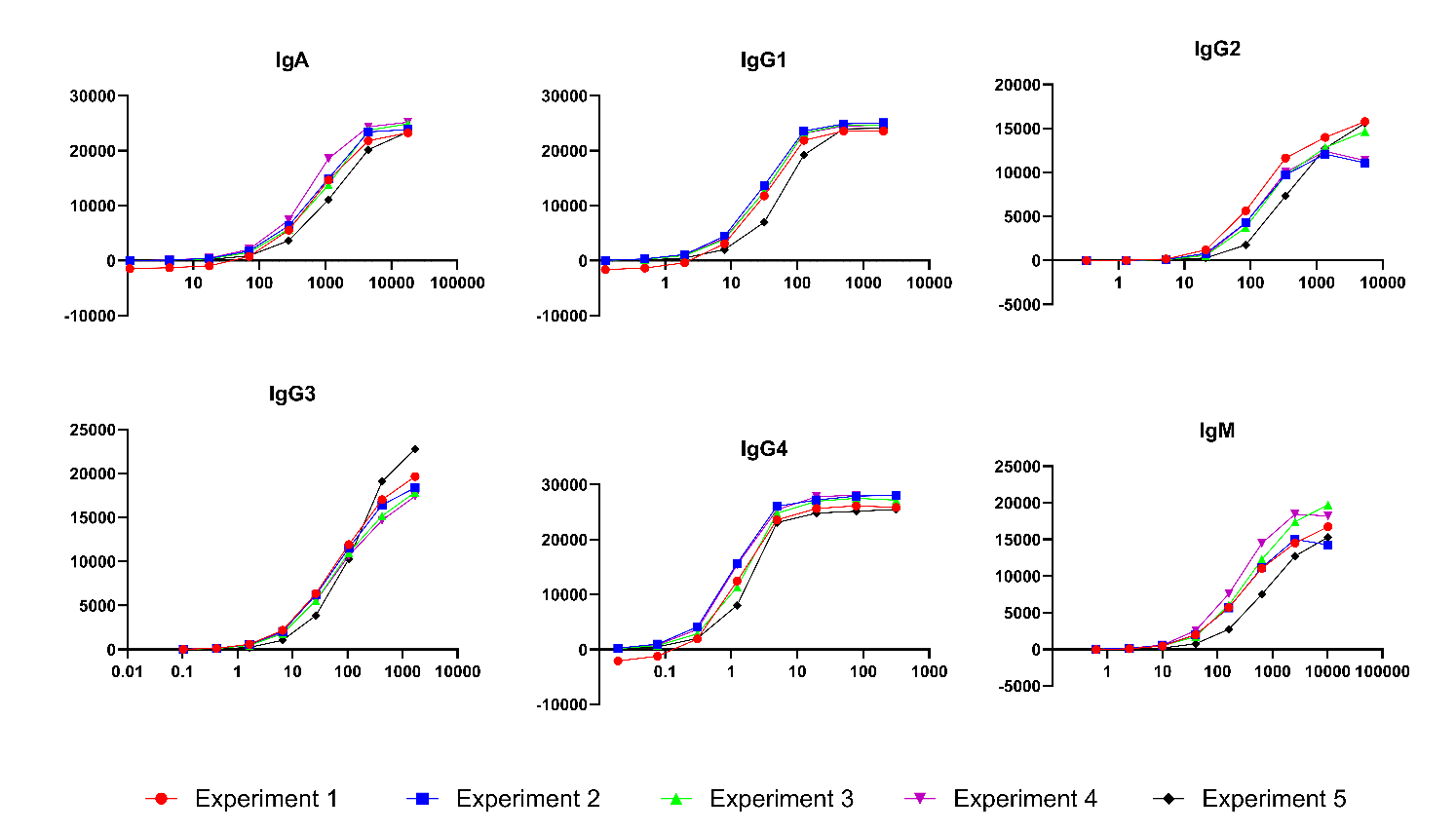


# Supplementary figure 1. Standard curves for the immunoglobulin isotyping assays run on different days.


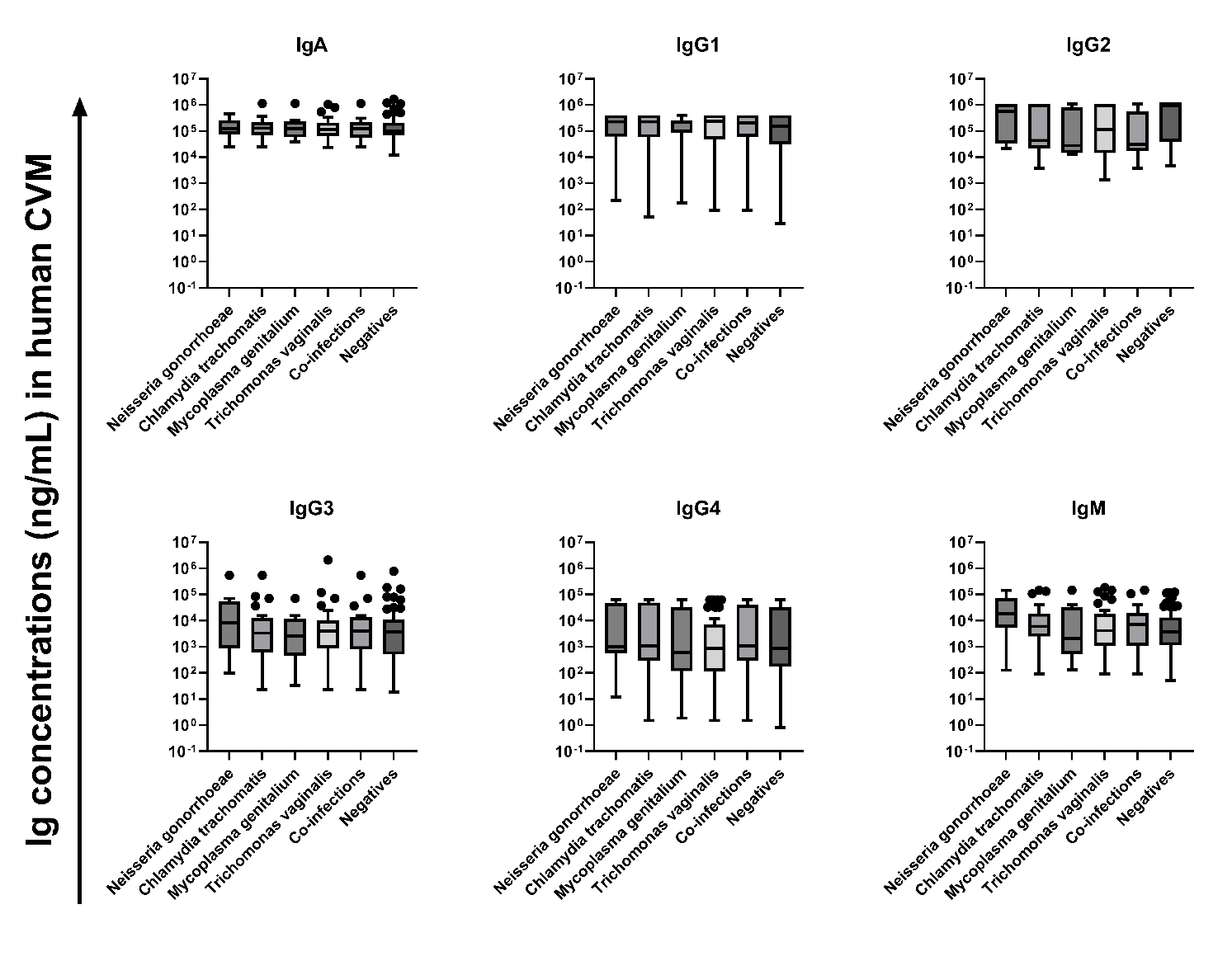


**Supplementary figure 2:** Comparison of the tested immunoglobulin concentrations (ng/mL) among the different tested STIs. Co-infections included participants with at least two of the tested STIs. Statistical test used for comparison among the groups was Kruskal-Wallis test with Dunn’s multiple comparison at 95% confidence interval.


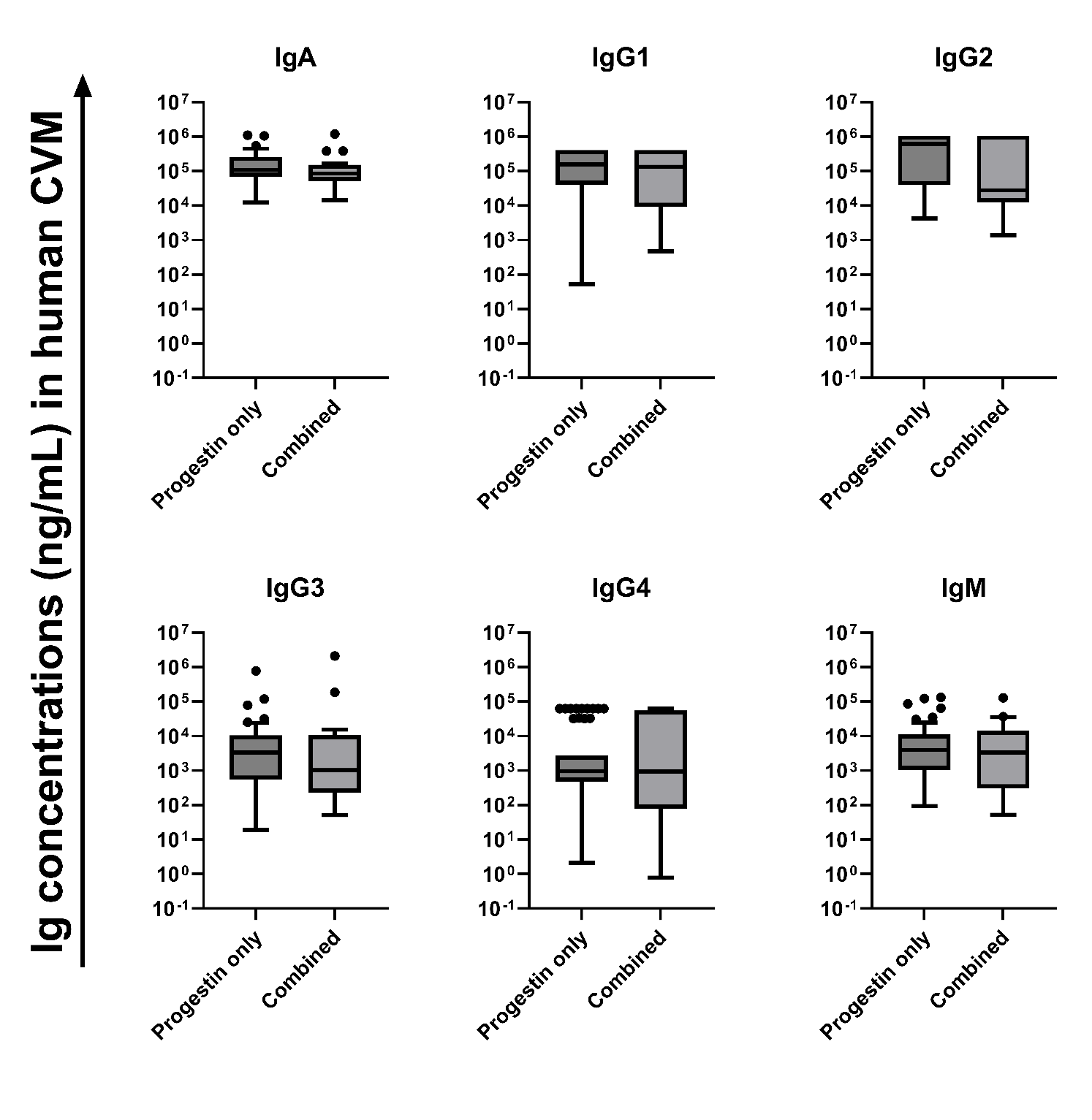


**Supplementary figure 3:** Comparison of the tested immunoglobulin concentrations (ng/mL) based on the type of hormonal contraceptive used. Statistical test used for comparison between the groups was Mann-Whitney U t test at 95% confidence interval.

## Supplementary tables

**Supplementary table 1:** The effect sizes (unstandardized β coefficients) and p values for the multiple linear regression analysis of the tested immunoglobulins adjusted for the listed variables. The significant β coefficients and p values are highlighted in red.

**Supplementary table 2:** The effect sizes (standardized β coefficients) and p values for the multiple linear regression analysis of the tested immunoglobulins adjusted for the listed variables. The significant β coefficients and p values are highlighted in red.

**Supplementary table 3:** The effect sizes (unstandardized β coefficients) and p values for the multiple linear regression analysis of the tested T/F HIV-1 virions mobility (MSD) with the measured immunoglobulins (ng/mL). The significant β coefficients and p values are highlighted in red.

**Supplementary table 4:** The effect sizes (standardized β coefficients) and p values for the multiple linear regression analysis of the tested T/F HIV-1 virions mobility (MSD) with the measured immunoglobulins (ng/mL). The significant β coefficients and p values are highlighted in red.
